# Whole-Genome Sequence of Clostridium botulinum A2B3 87, a Highly Virulent Strain Involved in a Fatal Case of Foodborne Botulism in Italy

**DOI:** 10.1128/genomeA.00237-15

**Published:** 2015-03-26

**Authors:** Francesco Giordani, Silvia Fillo, Anna Anselmo, Anna Maria Palozzi, Antonella Fortunato, Bernardina Gentile, Valentina Pittiglio, Ferdinando Spagnolo, Fabrizio Anniballi, Alfonsina Fiore, Bruna Auricchio, Dario De Medici, Florigio Lista

**Affiliations:** a Histology and Molecular Biology Section Army Medical and Veterinary Research Center, Rome, Italy; b National Reference Center for Botulism, Department of Veterinary Public Health and Food Safety, Istituto Superiore di Sanità (ISS), Rome, Italy

## Abstract

Here, we report the genome sequence of a rare bivalent strain of Clostridium botulinum, A2B3 87. The strain was isolated from a foodborne botulism case that occurred in Italy in 1995. The case was characterized by rapid evolution of the illness and failure of conventional treatments.

## GENOME ANNOUNCEMENT

Clostridium botulinum is an anaerobic spore-forming bacterium capable of synthesizing the botulinum neurotoxin (BoNT), a powerful and highly lethal poison (1). BoNTs are the only toxins which are tier 1 select agents (select agents and toxins lists are available at http://www.selectagents.gov). The intoxication by BoNT causes a neuromuscular paralysis produced by the block of peripheral cholinergic synapses. Botulism occurs from ingestion of preformed BoNT within contaminated food (food-borne) or by infections with bacterial spores and consequently toxin formation *in situ* (intestinal or wound) (1). The C. botulinum taxon has been divided into four groups (I to IV), as demonstrated by rRNA 16S gene comparison, amplified fragment length polymorphism (AFLP), and other techniques (2–4). On the basis of their serological activity, Clostridium botulinum strains are classified by 8 serotypes of BoNT (A to H), which are further divided into subtypes (A1 to A5, B1 to B7, E1 to E9, F1 to F7). Sixteen C. botulinum strains were sequenced and analyzed and several draft assemblies are available. These data show significant differences between the four groups (5–7).

A2B3 87 C. botulinum strain was isolated from a clinical case of foodborne botulism that occurred in a 76-year-old woman in Italy in 1995. The patient died 4 days after the hospital admission after being treated with the polyvalent antiserum and supported by respiratory aid. The bacterium was isolated in a sample of canned macrobiotic food based on “seitan,” a traditional Eastern recipe (8). The strain was found to produce both A and B toxin serotypes (ratio 10/1) (8).

The A2B3 87 genome was sequenced with the Roche 454 GS FLX Titanium and Illumina MiSeq platforms. From the 454 sequencing, a ~25× coverage was obtained (103,330,725 total sequenced bases, 272,724 total reads), while MiSeq sequencing reached ~246× coverage (970,106,826 total sequenced bases, 3,350,829 total paired reads). Illumina reads were used to cover the gaps in the 454 sequencing assembly and to correct the homopolymers length inaccuracies produced by 454 sequencing (9). The final draft assembly, with a G+C content of 27.9%, consists of 13 contigs. The 11 representing the chromosomal sequence are 3,847,714 bp long, while the 2 that constitute the plasmids are 275,568 and 45,268 bp long. The chromosomal gaps are caused by unresolved repeated sequences: the nine copies of the rRNA genes operon (total length of ~43 kb) and the two copies of the beta-*N*-acetyl-glucosamidase gene.

The bigger plasmid contains the two (A and B) BoNT genes. The BoNT/A gene is an A2 subtype, with a similarity of 99.85% (2 amino acid different) with A2 Kyoto BoNT sequence (YP_002803127.1) (3). BoNT/B is a B3 subtype but shows a considerable number of amino acid mutations compared to the other B3s; the similarity with CDC 795 (EF028400.1) is 98.22% and there are 21 different amino acids. More studies are needed to really understand the role played by the amino acid substitutions in this BoNT sequence. Moreover, the smaller plasmid showed no homologies with any other plasmid sequenced to date.

### Nucleotide sequence accession number.

The genome sequence of C. botulinum A2B3 87 is available in DDBJ/EMBL/GenBank under the accession no. AUZB00000000.

## References

[1] Arnon SS, Schechter R, Inglesby TV, Henderson DA, Bartlett JG, Ascher MS, Eitzen E, Fine AD, Hauer J, Layton M, Lillibridge S, Osterholm MT, O’Toole T, Parker G, Perl TM, Russell PK, Swerdlow DL, Tonat K, Working Group on Civilian Biodefense 2001 Botulinum toxin as a biological weapon: medical and public health management. JAMA 285:1059–1070. doi:10.1001/jama.285.8.1059. 11209178

[2] Collins MD, East AK 1998 Phylogeny and taxonomy of the foodborne pathogen *Clostridium botulinum* and its neurotoxins. J Appl Microbiol 84:5–17. doi:10.1046/j.1365-2672.1997.00313.x. 15244052

[3] Hill KK, Smith TJ, Helma CH, Ticknor LO, Foley BT, Svensson RT, Brown JL, Johnson EA, Smith LA, Okinaka RT, Jackson PJ, Marks JD 2007 Genetic diversity among botulinum neurotoxin-producing clostridial strains. J Bacteriol 189:818–832. doi:10.1128/JB.01180-06. 17114256 PMC1797315

[4] Olsen JS, Scholz H, Fillo S, Ramisse V, Lista F, Trømborg AK, Aarskaug T, Thrane I, Blatny JM 2014 Analysis of the genetic distribution among members of *Clostridium botulinum* group I using a novel multilocus sequence typing (MLST) assay. J Microbiol Methods 96:84–91. doi:10.1016/j.mimet.2013.11.003. 24246230

[5] Sebaihia M, Peck MW, Minton NP, Thomson NR, Holden MTG, Mitchel WJ, Carter AT, Bentley SD, Mason DR, Crossman L, Paul CJ, Ivens A, Wells-Bennik MHJ, Davis IJ, Cerdeño-Tárraga AM, Churcher C, Quai MA, Chillingworth T, Feltwell T, Fraser A, Goodhead I, Hance Z, Jagels K, Larke N, Maddison M, Moule S, Mungall K, Norbertczak H, Rabbinowitsch E, Sanders M, Simmonds M, White B, Whithead S, Parkhill J 2007 Genome sequence of a proteolytic (group I) *Clostridium botulinum* strain hall A and comparative analysis of the clostridial genomes. Genome Res 17:1082–1092. doi:10.1101/gr.6282807. 17519437 PMC1899119

[6] Peck MW, Stringer SC, Carter AT 2011 *Clostridium botulinum* in the post-genomic era. Food Microbiol 28:183–191. doi:10.1016/j.fm.2010.03.005. 21315972

[7] Skarin H, Håfström T, Westerberg J, Segerman B 2011 *Clostridium botulinum* group III: a group with dual identity shaped by plasmids, phages and mobile elements. BMC Genomics 12:185. doi:10.1186/1471-2164-12-185. 21486474 PMC3098183

[8] Franciosa G, Fenicia L, Pourshaban M, Aureli P 1997 Recovery of a strain of *Clostridium botulinum* producing both neurotoxin A and neurotoxin B from canned macrobiotic food. Appl Environ Microbiol 63:1148–1150. 9055430 10.1128/aem.63.3.1148-1150.1997 PMC168405

[9] Balzer S, Malde K, Jonassen I 2011 Systematic exploration of error sources in pyrosequencing flowgram data. Bioinformatics 27:i304–i309. doi:10.1093/bioinformatics/btr251. 21685085 PMC3117331

